# Pulse Oximetry Desaturation in the Postoperative Recovery Room in Patients with Obesity and Diabetes Using GLP-1 Agonists: A Retrospective Observational Study

**DOI:** 10.7759/cureus.87774

**Published:** 2025-07-12

**Authors:** Kimberly L Skidmore, Hong Le, Veronica Segredo, Jolie A Boullion, Charles P Daniel, Van Smith, Giustino Varrassi, Sahar Shekoohi, Alan D Kaye

**Affiliations:** 1 Department of Anesthesiology, Louisiana State University Health Sciences Center, Shreveport, USA; 2 Department of Anesthesia and Perioperative Care, University of California San Francisco, San Francisco, USA; 3 School of Medicine, Louisiana State University Health Sciences Center, Shreveport, USA; 4 Department of Pain Medicine, Fondazione Paolo Procacci, Rome, ITA

**Keywords:** ambulatory surgery, desaturation, diabetes mellitus, gastroparesis, glucagon-like peptide-1, laryngeal mask airway, morbid obesity, subclinical aspiration pneumonia

## Abstract

Background and objectives: Glucagon-like peptide-1 agonists (GLP-1), obesity, and diabetes may delay gastric emptying in some settings. We tested three hypotheses. First, the duration of GLP1-hold is associated with a larger difference between pulse oximetry at room air immediately preoperatively and one hour postoperatively, just prior to discharge home (DSpO2), as a marker of clinically important atelectasis and/or micro-aspiration. Second, we tested the hypothesis that the duration of GLP1-hold affects fasting glucose. Third, we tested the hypothesis that DSpO2 is linked to morbid obesity.

Materials and methods: In this retrospective observational cohort study, we screened the cohort of all 1571 patients undergoing urologic ambulatory surgery in one stand-alone center between September 2023 and September 2024. The inclusion criteria were diabetics using GLP-1 within 30 days, creatinine below 2 mg/dL, and age between 18 and 80 years. The outcomes of fasting glucose and DSpO2 were compared between the groups holding GLP-1 longer and shorter than seven days. The outcome DSpO2 was also compared between the groups with and without morbid obesity, defined generally as a body mass index (BMI) of over 35 kg/m² in the presence of one major comorbidity. All of our study subjects had diabetes mellitus, a major comorbidity.

Results: Among all 107 subjects, 56% had laryngeal mask airways and 9% endotracheal tubes. GLP-1 was held 12 ± 8 days (mean ± SD). DSpO2 was 1.5% ± 1.9 with short GLP-1-hold versus 1.8% ± 2.0 with long GLP-1-hold (p=0.41). The mean glucose was 130 mg/dL ± 49 with short GLP1-hold versus 138 mg/dL ± 39 with long GLP-1-hold (p=0.69). DSpO2 was 2.29% ± 1.78 with morbid obesity (N=38) versus 1.43% ± 1.99 without (N=69, p=0.0254, unpaired two-tailed t-test). Patients with morbid obesity showed a trend toward twice the incidence (at 24%) for DSpO2 > 4% (chi-square, p=0.14).

Conclusions: It may be prudent to protect the airway with endotracheal intubation for patients with morbid obesity and diabetes using GLP-1, especially in the lithotomy position.

## Introduction

Frank aspiration of stomach contents is a dreaded but rare complication of anesthesia. Investigational studies use two main methods to assess risk. One method to quantify residual fasting gastric volume (GV) is endoscopy, and the other is ultrasound. In a retrospective analysis of 404 endoscopies, in which 33 patients used glucagon-like peptide-1 agonists (GLP-1), only one patient suffered pulmonary aspiration [1]. Patients using GLP-1 were several times more likely to exceed a GV of 0.8 mL/kg [1]. Evidence that a drug induces an effect requires a dose-response curve; however, they found an all-or-none response, where GLP-1 discontinued for various durations (expected to result in corresponding drug levels) was associated with elevated GV. More importantly, the exact GV cutoff value relating to clinically relevant aspiration is multifactorial. For example, patients who described symptoms of delayed gastric emptying, such as nausea, vomiting, abdominal distension, and dyspepsia, were several times more likely to exceed a GV of 0.8 ml/kg [1]. Sen et al. used ultrasound to study a higher cutoff value, 1.5 ml/kg, in twice as many subjects [2]. Similarly, Sen demonstrated that GLP-1 use led to several times the risk of elevated GV. GLP-1 increases GV to variable degrees, unrelated to the duration of holding the medication (GLP1-hold), making it difficult to optimize this timing before anesthesia [1,2]. One of the many reasons for the variability is that, pharmacokinetically, to eliminate the effects of GLP-1, they should be discontinued for at least four half-lives [3]. Another reason for the variability is that vagal efferent nerves exhibit tolerance, manifested in lower GV after the initial escalation of dose [3]. In this regard, clinical scenarios commonly describe the period during which an initial escalation of dose is the highest risk for increased GV.

Obesity and diabetes contribute to aspiration risk by increasing GV [2]. Diabetes mellitus, type I and, to a lesser extent, type II, when paired with uncontrolled glucose, is associated with a 50% higher GV [4]. Additional factors such as pain, opioids, antacids, and cardiac medications increase GV [4]. Obesity-associated gastroesophageal reflux aggravated by obstructive sleep apnea is particularly severe when supine [5]. Perioperative frank aspiration of stomach contents is a rare complication, and risk factors are difficult to assess in a small study [6]. Conversely, subclinical micro-aspirations, which are more benign and frequent, are amenable to study. Therefore, we aimed to quantify the severity of micro-aspiration in obese diabetics using GLP-1, especially when ambulatory surgery requires supine and lithotomy positions.

To further reduce the complexity and sample size of our study, we targeted a population without a protected airway. Despite newer-generation laryngeal mask airways (LMAs) that help prevent gastric distension in obese patients, pulse oximetry desaturation often dictates conversion to endotracheal intubation [7]. Another example of conversion to a protected airway occurs in many patients using GLP-1 when GV prevents endoscopy imaging until contents are suctioned [8]. Nocturnal micro-aspiration is common in obesity due to gastroesophageal reflux [5,9]. Sustained oxygen saturation (SpO2) above 92% is a prerequisite for discharge from ambulatory surgery centers and alerts clinicians to a clinically important magnitude of aspiration [10]. Due to the lack of accepted markers for micro-aspiration and markers for atelectasis, we chose to measure the more clinically relevant difference between the baseline preoperative SpO2 and postoperative SpO2 at room air just prior to discharge home (DSpO2), one hour after surgery. Using DSpO2 to screen for micro-aspiration is based on several prospective trials documenting swallowing studies after strokes, which showed a correlation between the amount of liquid aspirated and DSpO2 [11,12]. While DSpO2 is controversial as a solo indicator of micro-aspiration, video fluoroscopy in 80 patients demonstrated that DSpO2 > 2% is an acceptable screening tool for initial clinical diagnosis (11). This tool is sensitive but not specific for micro-aspiration after stroke. The sensitivity of DSpO2 > 2% was 100% for simultaneous radiologic images, although the specificity was low [11,12]. Atelectasis, i.e., alveolar collapse, is a related phenomenon physiologically and is the most common explanation for DSpO2 perioperatively. Regardless of the etiology of DSpO2 (micro-aspiration or atelectasis), desaturation is a critical outcome that prevents discharge home after ambulatory surgery. Starting at a lower baseline SpO2 and older age were also risk factors for micro-aspiration after stroke [12]. Accuracy remained excellent for DSpO2 > 2% at both 2 and 10 minutes after aspiration after stroke; however, after the resolution of bronchospasm and atelectasis, SpO2 normalized [11,12]. Quick resolution is expected in those studies, given that the types of liquid aspirated were less toxic to the lungs than gastric secretions.

The optimal duration of preoperative GLP-1 discontinuation is controversial due to variable effects not only on gastric motility but also on hyperglycemia. We therefore tested three hypotheses. First, we tested the hypothesis that clinically relevant atelectasis and/or micro-aspiration, using DSpO2 as a marker, are related to GLP-1-hold duration. Second, we tested the hypothesis that GLP1-hold duration correlates with fasting hyperglycemia. Third, we tested the hypothesis that morbid obesity is related to DSpO2 in this high-risk group for aspiration and atelectasis, given the GLP-1 use, diabetes, obesity, LMA use, and lack of endotracheal tubes.

## Materials and methods

**Figure 1:**
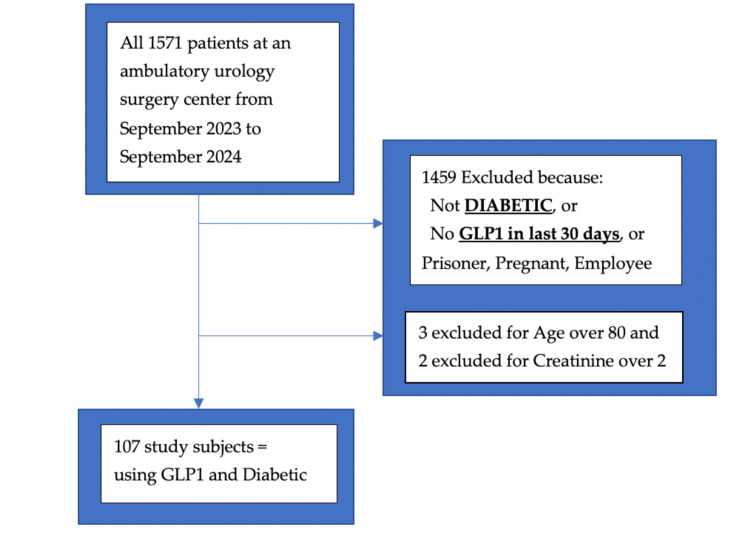
A flowchart outlining the inclusion and exclusion of participants in the study

This retrospective observational study was approved (protocol number STUDY00002715) by the Institutional Research Ethics Review Board of the Louisiana State University Health, Shreveport, LA. In accordance with the Helsinki Declaration and with minimal risk to privacy with anonymized data, the Institutional Review Board (IRB) waived informed consent from each study participant (number STUDY00002715). We retrospectively screened all 1,571 electronic medical records from one ambulatory urologic surgical center between September 2023 and September 2024 (Figure 1). We applied the STROBE checklist criteria recommended for retrospective study design (Appendix A)

We did not record the duration or type of surgery. The typical surgical procedures requiring deep sedation were mostly prostate biopsies, and those requiring general anesthesia with LMA or endotracheal intubation, at the discretion of the anesthesiologist, were mostly cystoscopies for renal calculi. No neuraxial anesthesia was performed in the present investigation. The clinicians directly providing anesthesia were the same two certified registered nurse anesthetists employed by the facility, under the supervision of various anesthesiologists. The two inclusion criteria were a diagnosis of diabetes and use of GLP-1 agonists within 30 days. Patients were identified by manually searching for the word “diabetes” and each of the following five GLP-1 medications: Ozempic, Rybelsus, Trulicity, Mounjaro, and Victoza, using EPIC, our electronic medical record system. Exclusion criteria were students, employees, incarcerated patients, mentally impaired patients, patients under 18 years old, pregnant patients, patients over 80 years old, and patients with creatinine >2 mg/dL. The final study group comprised 107 patients (Figure 1). Baseline demographics collected were GLP-1s taken (drug, dose, timing of last dose), weight, height, BMI, age, insulin dose, other diabetes medications, hemoglobin A1c (HbA1c), creatinine, and preoperative fasting glucose (pre-Glc) which was measured by a finger stick and point-of-care glucometer in the immediate preoperative minutes. We recorded the baseline preoperative SpO2 at room air, measured immediately prior to transfer from the preoperative holding room to the operating theater, and the final postoperative SpO2 recorded in EPIC, just prior to removal of the probe, upon discharge home. SpO2 was monitored with TruSignal reusable probes (General Electric, Finland Oy) and B4 monitors (General Electric Healthcare). We also recorded the anesthesia technique that was used: general anesthesia with the use of an endotracheal tube (ETT), general anesthesia with an LMA, or native airway with deep sedation (DS) without ETT or LMA.

Anonymized data were manually extracted and entered into Microsoft Excel (Microsoft Corp., Redmond, WA) by two investigators, K.S. and H.L., in alternating months. Microsoft Excel 14.4.3 unpaired two-tailed t-tests displayed fasting glucose in the group with preoperative hold of GLP-1 agonist longer than seven days and the group shorter than seven days. We applied t-tests to investigate whether GLP-1 hold duration affected the difference between pulse oximetry at room air preoperatively and postoperatively, just prior to discharge home (DSpO2). We also used t-tests to compare whether a BMI of greater than 35 kg/m² was associated with increased DSpO2. The chi-square test and Fisher’s exact test (when the number in a cell was five or less) were used to determine if severe DSpO2 (> 4% or > 5%) was more common with BMI > 35 kg/m^2^.

Excel generated means +/- standard deviations and t-test p-values. Excel allowed Fisher’s exact test one-tailed p-values by the HYPGEOM.DIST (value in first cell, total column count, total row count, total sample size, TRUE) syntax. R Core Team software generated figures with “R: A Language and Environment for Statistical Computing” (v4.4.2; 2024, by R Foundation for Statistical Computing, Vienna, Austria, https://www.R-project.org/). Pearson's Moment Correlation Coefficient (r) lines were drawn for all raw data on scatterplots: duration of GLP1-hold versus fasting glucose (pre-Glc), duration of GLP1-hold versus SpO2 prior to discharge to home, and BMI versus SpO2 prior to discharge home. R software provided chi-square and sample size calculations after data collection.

## Results

Table 1 describes baseline characteristics such as BMI, age, and HbA1c. Table 2 shows which GLP-1 was prescribed and at what dose. The most common medication was Ozempic (mean 1.3 mg weekly). Rybelsus and Victoza were prescribed every day (QD), while the others were administered as once-a-week injections (qWeek). Table 3 displays which anesthetic route was chosen. Table 4 lists the oral diabetic agents present in over half of these patients. Insulin use was present in 27 patients at a mean dose of 45 units/day.

**Table 1:** Baseline characteristics

Demographics	Mean	Standard deviation
BMI (kg/m^2^)	32.7	5.8
Age (years)	62	11
HbA1c (%)	7.1	1.7

**Table 2:** GLP-1 agonists taken by the study subjects. Dose in milligrams (mg); N: number of patients; QD: every day; QWeek: every week; Q1 is the first quartile and Q3 is the third quartile.

Parameters	Ozempic (qWeek)	Mounjaro (qWeek)	Rybelsus (qD)	Trulicity (qWeek)	Victoza (qD)
N	52	27	4	17	7
Mean dose mg	1.3	6.3	7.8	2.1	1.5
Minimum mg	0.25	0.75	0.75	0.6	7
Q1 mg	0.5	2.5	0.75	1.8	7
Median mg	1	6.25	1.125	1.125	7
Q3 mg	2	7.5	3	3	12.25
Maximum mg	8	15	4.5	4.5	14
% of subjects with missing data for dose	21	3.7	0	5.8	28

**Table 3:** Anesthesia mode chosen GA: general anesthesia; ETT: endotracheal tube; LMA: laryngeal mask airway; DS: deep sedation without ETT or LMA

Anesthesia mode	Number of subjects	Percentage (%) of subjects
GA with ETT	10	9.3
GA with LMA	60	56
DS	37	34.6

**Table 4:** Other medications

Other medications	Number of subjects	Percentage (%) of subjects
Insulin	27 (in the 16 with dose data, mean dose 45 units/day)	25
Metformin	43	40
Other agents	45 (glipizide, pioglitazone, glimepiride, sitagliptin, dapagliflozin, empagliflozin)	42

Table 5 shows the incidence of DSpO2 ≥ 4%, which increased from 13% to 24% in the morbidly obese cohort (BMI > 35 kg/m²), with a chi-square p-value of 0.14. Sample size calculations estimate that 388 subjects would have been required to reach an alpha of 0.05 and a beta of 0.20. Table 6 shows the incidence of DSpO2 ≥ 5%, which increased from 4.3% to 7.9% in the morbidly obese cohort (BMI > 35 kg/m^2^), with a chi-square p-value of 0.36. Sample size calculations estimate that 1,386 subjects would have been required to reach an alpha of 0.05 and a beta of 0.20.

**Table 5:** Delta SpO2 ≥ 4% in patients with BMI > 35 kg/m2 The incidence of Delta SpO2 ≥ 4% rose from 13% with BMI ≤ 35 kg/m^2^ to 24% in patients with BMI > 35 kg/m^2^. N is the number of subjects as values in cells. The chi-square test was nonsignificant. SpO2: oxygen saturation

Chi-square p = 0.14	Delta SpO2 ≥ 4% displayed as N (%)	Delta SpO2 < 4% displayed as N
BMI ≤ 35 kg/m^2^	9 (13%)	60
BMI > 35 kg/m^2^	9 (24%)	29

**Table 6:** Delta SpO2 ≥ 5% in patients with BMI > 35 kg/m2. The incidence of Delta SpO2 ≥ 5% rose from 4.3% with BMI ≤ 35 kg/m^2^ to 7.9% in patients with BMI > 35 kg/m^2^. One-tailed Fisher’s exact test p-value: 0.36. SpO2: oxygen saturation

Fisher’s exact p = 0.36	Delta SpO2 ≥ 5% displayed as N (%)	Delta SpO2 < 5% displayed as N
BMI ≤ 35 kg/m^2^	3 (4.3%)	66
BMI > 35 kg/m^2^	3 (7.9%)	35

Mean DSpO2 was 0.86% greater, at 2.29 % ±1.78, in the 38 patients with BMI > 35 kg/m^2^, versus 1.43 ±1.99 in the 69 patients with BMI ≤ 35 kg/m^2^ (mean ± SD, p=0.0254, unpaired two-tailed t-test) (Table 7).

**Table 7:** Mean Delta SpO2 and BMI. Delta SpO2 is the difference between preoperative baseline SpO2 at room air and SpO2 just prior to discharge. Mean delta SpO2 was 2.29 ± 1.78% in the group with BMI > 35 kg/m² versus 1.43 ± 1.99 with BMI ≤ 35 kg/m² (p=0.0254, unpaired two-tailed t-test). SpO2: oxygen saturation; SD: standard deviation; N: number of patients

Two-tailed t-test *p=0.0254	Delta SpO2 mean	Standard deviation (N)
BMI ≤ 35 kg/m^2^	1.43 %	1.99 (69)
BMI > 35 kg/m^2^	2.29 %	1.78 (38)
Difference of mean Delta SpO2 between BMI ≤ 35 kg/m^2^ and BMI > 35 kg/m^2^ groups	0.86 %	

**Figure 2:**
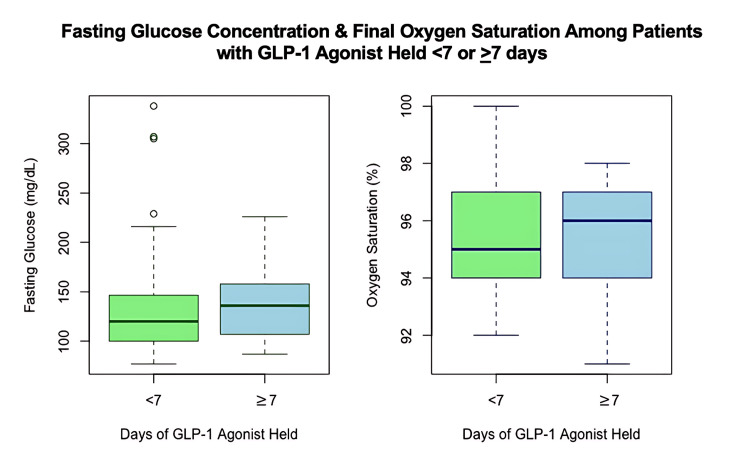
Box plot on left: The Y axis shows fasting glucose concentration (mg/dL). The mean glucose was 130 mg/dL ± 49 in the group with < 7 days GLP1-hold (N=29 had available pre-Glc data) versus 138 mg/dL ± 39 in the group with ≥ 7 days GLP1-hold (N=67 had available pre-Glc data) (two-tailed unpaired t-test p=0.69). Box plot on the right: The Y axis shows the final (just prior to discharge home) oxygen saturation. The first box represents subjects with < 7 days of GLP1-hold, with data available in all 31 patients. The second box represents subjects with ≥ 7 days of GLP-1 hold, with data available in all 76 patients. The central line in the box plot is the median, the top of the box is the third quartile, the bottom of the box is the first quartile, and the whiskers are 1.5 times the interquartile range. pre-Glc: fasting glucose from a fingerstick glucometer immediately preoperatively

**Figure 3:**
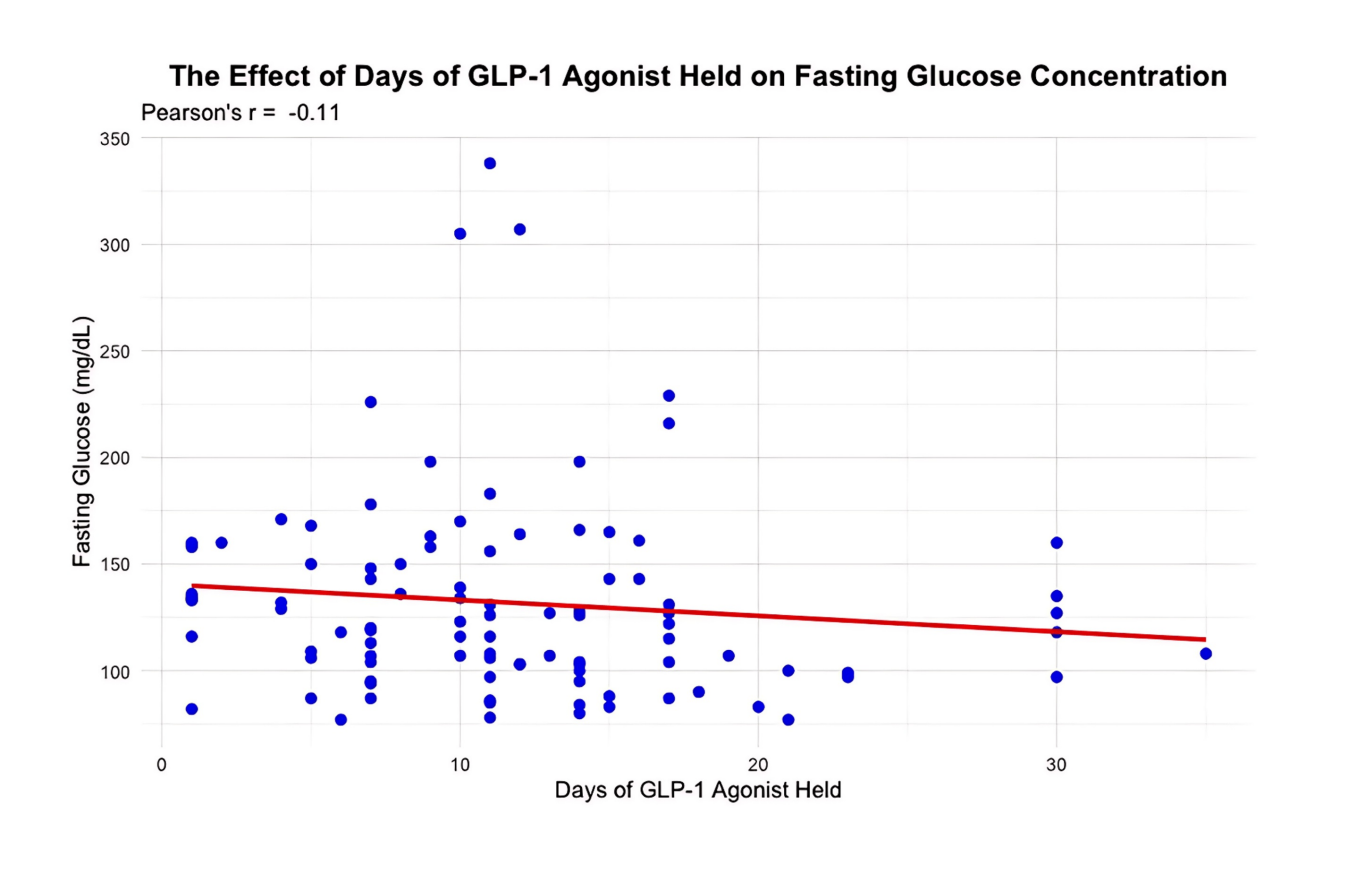
Correlation coefficient by the R statistical program The X axis is the number of days the GLP-1 medication was held. The Y-axis is the fasting glucose in mg/dL. The R value is the Pearson moment correlation coefficient, R.

**Figure 4:**
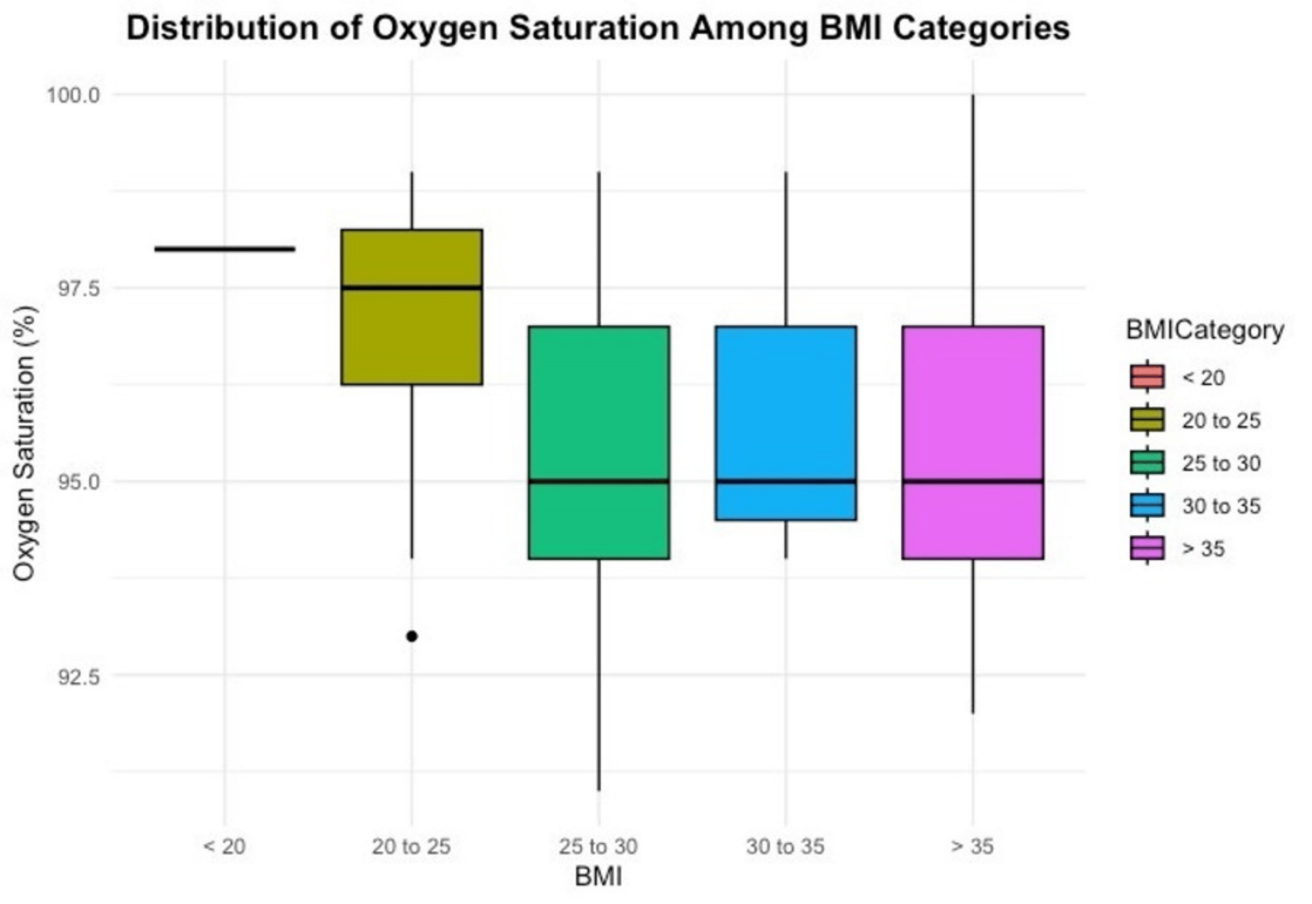
Distribution of oxygen saturation among BMI categories The Y axis is the final oxygen saturation recorded just prior to discharge from the recovery room to home. The x-axis is the BMI (weight in kg divided by meters squared of height).

GLP-1s were held for 12 days ± 8 (mean ± SD) in all patients. The pre-Glc was 130 mg/dL ± 49 (mean ± SD) in the group with short GLP1-hold (<7 days) versus 138 mg/dL ± 39 (mean ± SD) in the group with long GLP1-hold (≥ 7 days). Data for pre-Glc were available in 29 of the short-hold group and 67 of the long-hold group, p=0.69 t-test (Figure 2). The correlation coefficient between pre-Glc and GLP1-hold duration was r = -0.11 (Figure 3). DSpO2 was 1.5% ± 1.9 (mean ± SD) in patients with short GLP1-hold versus 1.8% ± 2.0 in patients with long GLP1-hold, with data available in all 31 patients in the short GLP1-hold group and 76 patients in the long GLP1-hold group, p=0.41. Figure 4 shows the final SpO2 prior to discharge home among various categories of BMI.

## Discussion

In this retrospective observational study, we investigated a novel marker of perioperative micro-aspiration, or a related event, alveolar collapse, i.e., atelectasis, DSpO2. DSpO2 has been validated as a sensitive initial screening tool pre-aspiration and 10 minutes post-aspiration after stroke using X-ray. We defined DSpO2 as the difference between SpO2 immediately preoperatively and just prior to discharge home, an hour after surgery. The Aldrete score was utilized along with common criteria for discharge home from ambulatory surgery. We targeted two risk factors in the inclusion criteria: a diagnosis of diabetes and the use of GLP-1 within 30 days. We found that only one variable, morbid obesity, was related to DSpO2. Morbid obesity was generally defined as a BMI above 35 kg/m² in the presence of any comorbidity (i.e., diabetes mellitus, which all study subjects had). We found that subjects who were morbidly obese had a significantly higher incidence of clinically relevant DSpO2, with a 0.86% greater mean DSpO2 (p = 0.02). Patients who were morbidly obese had a mean DSpO2 of 2.29%, well above the cutoff value of 2% established in the stroke literature to correlate with a witnessed aspiration on X-ray [11, 12]. Regardless of whether the DSpO2 was caused by micro-aspiration or atelectasis, desaturations are important because the patient will not be permitted to discharge home after ambulatory surgery below a final SpO2 of 92%. We identified a trend toward twice the risk of more serious DSpO2 > 4%, p = 0.14, in the morbidly obese group. Although this study was not powered to detect DSpO2 > 4%, only 281 more subjects would be required to reach significance. The three main etiologies of DSpO2 are atelectasis, bronchospasm, and aspiration. To eliminate the confounder etiologies of minor atelectasis and bronchospasm, we focused on the SpO2 reading just prior to discharge home. Major regions of atelectasis may cause desaturation to persist for several days and subsequently develop pneumonia. Whether related to atelectasis or aspiration is not as relevant as the depth and duration of clinical signs. A recent case illustrates the combination of aspiration and atelectasis in an infant following the insertion of an LMA. The CT scan in Appendix B demonstrates aspiration in the right upper lobe and a minor region of right lower lobe atelectasis. She was clinically asymptomatic, except for her SpO2 falling from 97% to 93% in the recovery room, returning to normal within 72 hours. An endotracheal tube does not reduce the overall risk of brief intraoperative DSpO2 commonly observed in obese patients with atelectasis [7]. However, if an ETT is present, atelectasis can be treated effectively with airway recruitment and positive end-expiratory pressure. When atelectasis is the culprit, airway recruitment maneuvers may involve sustained high-pressure ventilation intraoperatively, compounding aspiration risk in obese patients without an ETT if only using an LMA. Once in the recovery room, additional treatments may include routine incentive spirometry, ambulation, or bronchodilators. Analysis of discharge SpO2 in relation to preoperative SpO2 also eliminates some of the measurement bias from technical factors of pulse oximetry, such as skin color [13]. Dark skin tones result in an absolute 2% overestimate of SpO2 [13]. Another strength of our study is homogeneity within ambulatory urology patients, yielding similar risks but few comorbidities.

Our results confirm a recent study by Chen et al. showing that the duration of GLP1-hold does not correlate with postoperative pulmonary complications [10]. They studied 5,931 subjects and found no difference in respiratory complications between the group prescribed GLP-1 for diabetes and the group for weight loss [10]. A smaller endoscopy study delineated higher GV for diabetics [1]. Clinical outcomes such as aspiration and SpO2 matter more than GV fluctuation. We found no difference in DSpO2, regardless of GLP1-hold duration.

Although we studied fewer patients than Chen et al., our study is more sensitive for capturing micro-aspiration or atelectasis, since most of our patients (91%) did not have an ETT to protect the lungs [10]. Most of our patients (56%) had an LMA. More aspiration burden is predicted for obese patients because of the higher airway pressure leakage around the LMA into the stomach if pressure is above 25 cmH₂O [7]. Additionally, 56% of our subjects were placed in the lithotomy position during surgery. Lithotomy position by itself has been shown to increase aspiration risk [14]. Obesity likewise increases atelectasis and airway pressures, especially in the lithotomy position [8,9]. Therefore, caution is advised against LMA use in the lithotomy position if BMI is above 34 kg/m² and in the supine position if BMI is above 44 [14]. Airway resistance, gastroesophageal reflux, and lung compliance worsen throughout longer surgical procedures with an LMA [14].

The perioperative increase in aspiration risk of diabetic obese patients using GLP-1 has been recognized by the American Society of Anesthesiologists (ASA), which in some situations recommends delaying the procedure or, if surgery is urgent, intubating the trachea rapidly [15]. When it is challenging to quantify vague symptoms that introduce doubt about GV, the ASA recommends point-of-care ultrasound. GLP-1 agonists delay gastric emptying by a mean of 36 minutes, which would not be expected to elevate the risk of aspiration [16, 17]. A prospective study highlights exceptions, measuring large GV in 56% of patients using GLP-1 compared to only 19% in controls [2]. It is concerning that 19% of patients not using GLP-1 agonists had large GV. Our study sheds light on the often-ignored but ubiquitous issue of obesity. One option to improve safety is an increased duration of fasting [2, 6, 8, 17-21]. Another option is a 24-hour preoperative liquid diet [16,22,23]. Some reviews state that an LMA is relatively contraindicated in obesity, related to low pulmonary compliance and high aspiration risk [22]. In this regard, the role of neuraxial anesthesia and best practice strategies should be evaluated and determined for high-risk populations, including diabetics utilizing GLP-1 who are also morbidly obese.

We found no difference in preoperative glucose, regardless of GLP1-hold time. This is likely because glucose levels can be regulated by concomitant medications. Approximately half of our patients were taking oral hypoglycemic medications, and many were taking insulin. In structured healthcare environments with frequent patient engagement, outcomes may be improved by protocols that obviate GLP1 [24,25]. Research is lacking regarding the acute effects of GLP-1 cessation on perioperative blood glucose levels, although it has been shown that hemoglobin A1c begins to increase within two months [26, 27]. Over 70% of those using GLP-1 discontinue therapy within two years due to gastrointestinal symptoms [27]. Lower socioeconomic status (SES) is linked to discontinuation of therapy and obesity, but discontinuation is not linked to diabetes because its diagnosis often allows for reimbursement.

Limitations

Our study has two main limitations. Apart from being a small retrospective observational study, our ambulatory surgery center can only accommodate patients who are healthy, without major comorbidities such as a BMI > 50 kg/m², or uncontrolled states of pain, diabetes, anemia, or lung disease. It was not feasible to follow up beyond discharge from the recovery room. Future studies should differentiate outcomes and quantify minor or severe atelectasis or aspiration using chest X-rays, signs of pneumonia over the following month, and the total financial costs. Ideally, a multivariate analysis of thousands of patients would combine the highest risks into an index, including BMI, GLP-1, diabetes, LMA ventilator parameters, surgical types and positions, and duration of surgery, excluding patients with ETTs that allow protection from both aspiration and atelectasis.

## Conclusions

We demonstrated that morbid obesity is a risk factor for postoperative desaturation among the high-risk group of diabetic patients using GLP-1 without a protected airway and, therefore more likely to encounter atelectasis and/or micro-aspiration. The desaturation is independent of the duration of GLP1 hold time. Discontinuation of GLP-1 for more than one week does not increase preoperative fasting glucose levels, which may allow for prolonged GLP-1 hold time in efforts to prevent desaturation. At present, there are very few studies examining the association of prolonged desaturations with morbidly obese patients who are using GLP-1 and an LMA. Our investigation builds on previous studies that have shown increased BMI and LMA use are associated with desaturations, especially when in the lithotomy position. 

## Figures and Tables

**Figure 5:**
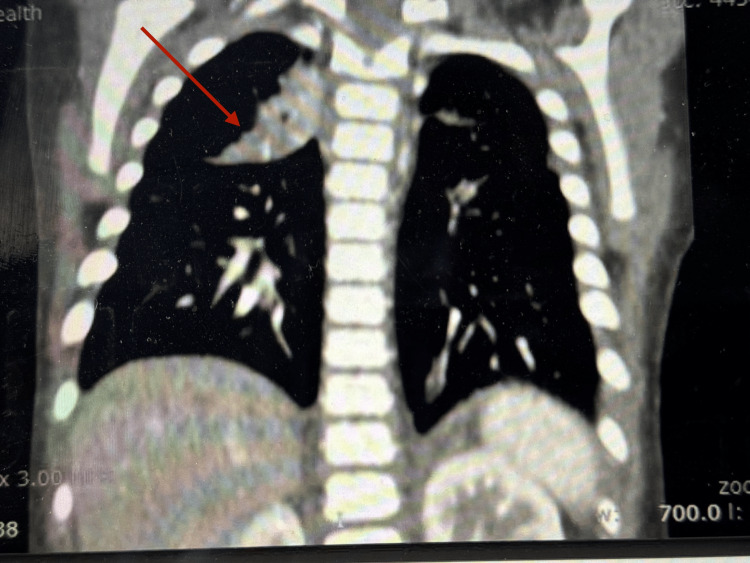
Clinical impact of DSpO2 = 4%. The red arrow points to acute pulmonary aspiration of gastric contents within the right upper lobe.

## Appendices

Appendix A 

Strengthening the Reporting of Observational Studies in Epidemiology (STROBE) Checklist

1. A retrospective observational study is in the title and commonly used terms. The abstract lists 3 hypotheses tested regarding GLP-1 and obesity affecting fasting glucose and desaturation.

2. The background is explained regarding atelectasis and micro-aspiration being at high risk in this subpopulation of diabetics using GLP-1 without endotracheal intubation.

3. The 3 hypotheses are listed in the abstract and introduction.

4. The design of the paper is a retrospective chart review of the entire cohort having surgery at an ambulatory urologic center. 

5. The data collection is listed in the hypotheses.

6. This is a cohort study. Inclusion and exclusion criteria are listed.

7. All outcomes are explained.

8. The assessment is explained.

9. Bias from probes is eliminated partially by following each individual's change in saturation.

10. The study size was explained in the sample size calculations.

11. BMI groups were examined according to desaturations, and thus a BMI cutoff of 35 kg/m² was arrived at.

12. All methods are described for the statistics. Missing data is listed in the table. The study is too small to analyze subgroups.

13. The flowchart of Figure 1 shows the inclusion and exclusion numbers of subjects.

14. The demographics are listed in the table.

15. The cohort is followed for all outcomes for the time period.

16. The study is too small to analyze confounders.

17. No additional analysis is done.

18. Key results are summarized.

19. The limitations are discussed as predominantly due to small sample size. Future studies should focus on the LMA and BMI over 35 groups to follow desaturation and etiologies.

20. The discussion relates prior studies to this study, especially regarding obesity and LMA, and desaturation.

21. This study should be generalizable to ambulatory surgery patients who are diabetic and use GLP-1. Future studies should analyze all LMA.

22. There was no funding for the data collection or preparation of the original manuscript.

Appendix B

Clinical Impact of DSpO2 = 4%
